# Sex differences in the knee orthopaedic injury patterns among recreational alpine skiers

**DOI:** 10.1186/s13102-020-00224-6

**Published:** 2020-12-04

**Authors:** Huijuan Shi, Yanfang Jiang, Shuang Ren, Xiaoqing Hu, Hongshi Huang, Yingfang Ao

**Affiliations:** 1 Institute of Sports Medicine, Peking University Third Hospital, Beijing Key Laboratory of Sports Injuries, Beijing, 100191 China; 2 School of Biological Science and Medical Engineering, Beijing Advanced Innovation Center for Biomedical Engineering, Beihang University, Beijing, China

**Keywords:** Anterior cruciate ligament, Skiing, Epidemiology, Gender

## Abstract

**Background:**

Although studies have reported the sex differences in injury patterns among recreational skiers, the findings are still conflicting. This study aims to analyse the sex differences of orthopaedic knee injuries that occurred during alpine skiing.

**Methods:**

A total of 306 recreational alpine skiers (125 females and 181 males) who sustained knee surgeries between June 2016 and December 2018 participated in this study. Age, height, weight, and physical activity level of the patients were recorded. The orthopaedic knee injury patterns were analysed based on the diagnosis given by the physicians.

**Results:**

Male skiers (17.13%) had a higher proportion of multiple knee ligament injuries than females (6.40%). The combined anterior cruciate ligament (ACL) and medial collateral ligament injury were the most common injury types in both females and males, with ACL injury being more prevalent for females (79.20%) than that in males (56.35%). The proportion of female skiers (17.6%) with vigorous-intensity activity level was significantly lower than that of males (30.9%). Female skiers had lower body height, body weight, and body mass index than male skiers (*P* <  0.001).

**Conclusions:**

ACL injury is the most common orthopaedic injury among both female and male knee-injured recreational skiers. The proportion of females with an ACL injury is higher than that of males, but the proportion of multiple knee ligament injuries is lower than that of males. More male recreational skiers have vigorous-intensity activity level habits in daily life than females.

## Background

Alpine skiing is a popular sport across the world that is associated with significant orthopaedic injuries [1, 2]. The rate of sustaining an injury while skiing was 2.8 per 1000 skier days on average from 2008 to 2010 [3]. A recent review reported that the overall injury incidence in snow sports was 3.49 per 1000 athlete-days [4]. The number of skiing participants has grown dramatically, with 11.5 million participating in skiing in 2010 in the United States alone [2].

Previous studies have reported that the most common injury site during skiing is the knee joint [5–7]. Knee injuries account for one-third of all adult alpine skiing injuries with decisive sex differences in knee injury rates [8]. An understanding of sex differences in the injury pattern of sports injuries is of utmost interest among researchers. Studies have shown that females are at higher risk for knee ligament injuries than males in competitive sports like basketball, soccer, and volleyball [9–12]. Although several studies have attempted to investigate sex differences in injury patterns among recreational skiers, the findings are still conflicting [13]. Studies by Ekeland et al. and Stevenson et al. reported that the percentage of knee injuries was almost twice as high for females compared to males in alpine skiers [14], and female alpine skier racers were 3.1 times more likely to have sustained an ACL injury, respectively than male racers [15]. However, some other studies reported no sex difference in the risk of knee and anterior cruciate ligament (ACL) injuries among skiers [16–18] and the skiing injury rates [7, 19]. The current studies suggest that the effect of sex on injury patterns is not apparent [13]. The literature controversy may be due to the varying definitions of injury among different studies [13]. In previous studies, the injury was often documented through the injury registration system. Particularized injuries cannot be accurately distinguished due to coding limitations in the registration system. For future studies, accurate diagnosis documented from clinicians with detailed injury information would be recommended.

Moreover, previous studies reported that the incidence of ACL injuries with concomitant injuries was higher than the isolated ACL injuries among alpine skiers [20]. Although a study by Girardi et al. reported that male skiers experienced more severe injuries than female skiers [21], our extensive review of the literature found no study reporting sex differences in detailed knee injury patterns and the combined injury diagnosis. In addition to injury patterns, males and females participate in different intensities of physical activity during adolescence [22]. A prospective study examined longitudinal changes in physical activity in a large cohort of adolescent girls and found that habitual leisure-time activity levels among girls aged nine to18 years declined substantially by the age of 18 [23]. Considering the significant sex disparity in ACL injury rate in high-school and collegiate students [24], the physical activity level might be one potential risk profile for sports injury. Win et al. reported that females were less likely to exercise regularly [25]. However, there is little known about the sex difference in physical activity habits among the recreational alpine skiers, which is needed to further investigate.

Therefore, the purpose of this study was to analyse the sex differences in orthopaedic knee injury patterns during alpine skiing and physical activity habits. We hypothesized that female recreational skiers would have a significantly higher proportion of the ACL injuries among the knee injuries compared with male skiers. We also hypothesized that female skiers would significantly lower the proportion of multiple ligament injuries among knee injuries than male skiers. We finally hypothesized that significant sex differences in the physical activity habits would exist among the injured skiers.

## Methods

### Patient selection

The use of human subjects in this study was approved by the Institutional Review Board of the hospital. All cases of knee surgeries performed in our institute between June 2016 and December 2018 were identified. This institute is the first and biggest sports medicine center in our country and is the only officially designated Athletic Injury Prevention and Treatment Center. Only the patients who got orthopaedic injuries that occurred during alpine skiing were included in the study. The patient should also be recreational skiers who did not ever participate in high-level competitions such as regional, state, or national competition. Orthopaedic injuries were defined as the musculoskeletal injuries that need to be repaired using orthopaedic surgeries. Diagnoses were made by experienced sports medicine physicians and were confirmed by arthroscopy. Patients aged younger than 18 years or the initial injury was re-injury were excluded from the study. A total of 306 participants were included in this study during the study period.

### Data recording and analysis

The patient demographics, including age, body weight, body height, and injury mechanism, were collected. Body mass index (BMI) was calculated as the body mass divided by the square of the body height. The injury mechanism of injury was divided into non-contact and contact. For patients to be classified into the contact group, a direct blow to the knee was applied during an injurious event. For example, the lower leg is hit by a tree, rock, or another skier while skiing. If there was no direct blow to the knee at the time of the injury, the mechanism was classified as non-contact [26]. Information on injury mechanisms was collected through a personal interview, the description of the injury situation as reported by the patient.

The orthopaedic injuries were also analysed based on the diagnosis given by the physicians. The number of patients reflects the number of separate diagnoses. The proportion of injuries was determined by dividing the patient number of a particular injury by the total number of patients. The single knee ligament injury was defined as having only one ligament injured; the multiple knee ligament injury was defined as having two or more ligaments injured.

Physical activity was assessed using the Global Physical Activity Questionnaires developed by the WHO [27]. Participants were asked about the intensity and frequency of physical activity under three domains (work, travel to and from places, and recreation) to classify their physical activity level. Vigorous-intensity activities were defined as activities that require a large amount of effort and causes rapid breathing and a substantial increase in heart rate. Moderate-intensity activities were defined as activities that require a moderate amount of effort and noticeably accelerate the heart rate. Sedentary behaviour was defined as sitting or reclining with low energy expenditure.

### Statistical analysis

Descriptive statistics for continuous variables normally distributed were reported as the means ± standard deviations. Independent t-tests were used to compare the demographics between sexes. Chi-squared tests and Fisher exact tests were performed to compare the injury mechanisms and physical activity levels between sexes. Chi-squared tests and Fisher exact tests were also performed to compare the injury patterns between sexes. A Type I error rate of 0.05 was selected as the indication of statistical significance. All statistical analyses were performed using the SPSS statistical analysis computer program package (SPSS, Chicago, IL, USA) version 16.

## Results

### Patient demographics

Among the 306 participants, 125 (40.7%) were female, and 181 (59.3%) were male during the study period (Table 1). Female and male skiers are similar in age (*P* = 0.081). Female skiers had significantly lower body height (*P* <  0.001), body mass (P <  0.001), and BMI (P <  0.001) as compared to the male skiers (Table 1). There was no significant difference in the injury mechanism between sexes (*P* = 0.446). 80.0% of the injured females and 84.0% of the injured males were non-contact injuries.

**Table 1:** Demographics of patients who sustained injuries from skiing

	Female (*n* = 125)	Male (*n* = 181)	Statistical Significance
Age, year	34.4 ± 10.6	36.4 ± 10.4	0.081
Body height, cm	163.3 ± 4.3	173.9 ± 6.2	< 0.001
Body weight, kg	58.5 ± 8.2	77.0 ± 12.6	< 0.001
BMI	21.9 ± 2.8	25.4 ± 3.4	< 0.001
Noncontact/contact, n	100/25	152/29	0.446

*Abbreviations*: *BMI* Body mass index

### Orthopaedic injury

The orthopaedic diagnoses were listed in Table 2. Statistical analysis showed that the injury type was significantly different between male and female skiers (*P* = 0.012). Compared with male skiers, female skiers had a higher proportion of the single knee ligament injury (*P* = 0.006) and a smaller proportion of the multiple knee ligament injury (*P* = 0.005) (Table 2). No significant sex differences were observed in the proportion of isolated meniscal injury (*P* = 0.305) (Table 2).

**Table 2:** The orthopaedic diagnoses in recreational skiers^*a*^

Injury type	Females		Males	
	n (percentage)	Noncontact/Contact	n (percentage)	Noncontact/Contact
Single knee ligament injury	106 (84.8%)	88/18	127 (70.17%)	105/22
MCL	3 (2.40%)	1/2	19 (10.50%)	12/7
LCL	0		1 (0.55%)	1/0
ACL	99 (79.20%)	84/15	102 (56.35%)	90/12
PCL	4 (3.20%)	3/1	5 (2.76%)	2/3
Isolated meniscal injuries	8 (6.40%)	4/4	19 (10.50%)	17/2
Medial	1 (0.80%)	0/1	8 (4.41%)	7/1
Lateral	6 (4.80%)	3/3	9 (4.97%)	8/1
Both Lateral and Medial	1 (0.80%)	1/0	2 (1.10%)	2/0
Multiple ligament injuries	8 (6.40%)	6/2	31 (17.13%)	27/4
Combined ACL and PCL	0		4 (2.21%)	4/0
Combined ACL and MCL	8 (6.40%)	6/2	16 (8.84%)	14/2
Combined ACL, MCL, and PCL	0		6 (3.31%)	5/1
Combined PCL and LCL	0		1 (0.55%)	0/1
Combined PCL and MCL	0		4 (2.21%)	4/0
Other injuries	3 (2.40%)	2/1	4 (2.21%)	3/1
Combined ACL and Intercondylar eminence fracture	0		1 (0.55%)	1/0
Combined MCL and Intercondylar eminence fracture	1 (0.80%)	1/0	0	
MPFL	1 (0.80%)	1/0	1 (0.55%)	1/0
Patellar Dislocation	1 (0.80%)	0/1	0	
Isolated cartilage injury	0		1 (0.55%)	1/0
Intercondylar eminence and Medial Tibial Plateau fractures	0		1 (0.55%)	0/1

^a^Values are expressed as number (percentages) within the relevant group

*ACL* anterior cruciate ligament, *MCL* medial collateral ligament, *PCL* posterior cruciate ligament, *LCL* lateral collateral ligament, *MPFL* medial patello-femoral ligament

For the single ligament injuries, the most common injury in females and males was ACL injury, representing 79.2% of all diagnoses in females and 56.4% of all diagnoses in males. Statistical analysis showed that the ACL injuries were more prevalent for females than that for males (*P* <  0.001) (Table 2). Besides, the male skiers demonstrated a significantly higher proportion of medial collateral ligament (MCL) injury than the female skiers (P <  0.001). For the multiple knee ligament injuries, the most common injury type in females and males was the combined ACL and MCL injury, which was also the only injury type in the multiple knee ligament injuries for female skiers (Table 2).

### Physical activity level

**Fig. 1:**
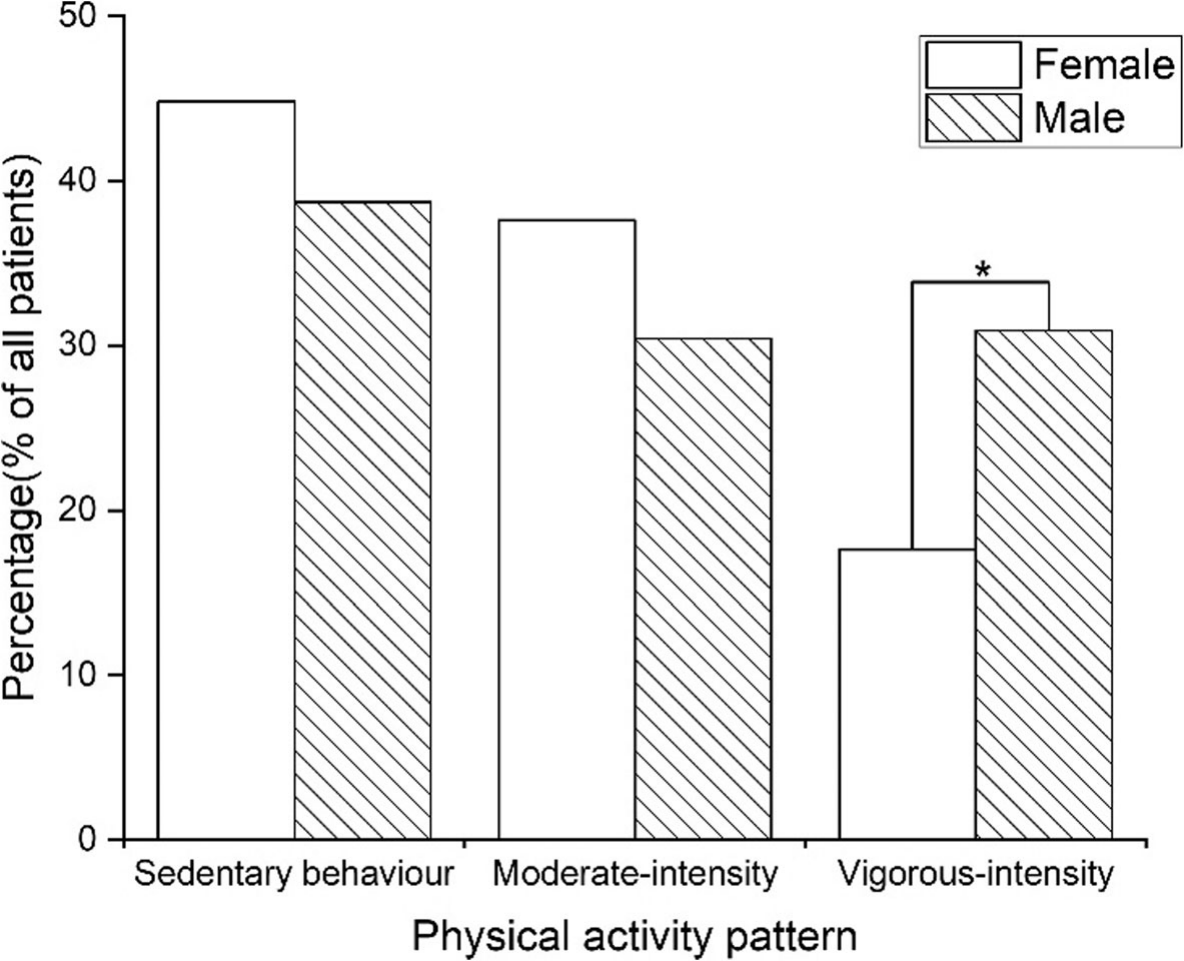
The difference in physical activity level between sexes. *Significantly lower proportion of female skiers with vigorous-intensity activity level than that of male skiers (*P* = 0.011)

There was a significant difference in physical activity levels between female and male skiers (*P* = 0.030) (Fig. 1). The proportion of patients with vigorous-intensity activity level was significantly lower in females (17.6%) than that in males (30.9%) (*P* = 0.011). No significant difference was found in the sedentary behavior level between female skiers (44.8%) and male skiers (38.7%) (*P* = 0.290). The proportion of moderate-intensity activity levels in females (37.6%) was similar to that in males (30.4%) (*P* = 0.218).

## Discussion

The results of this present study showed that an ACL injury was the most frequent knee injury diagnosis among recreational skiers. This result was also reported consistent with the literature that reported the most common orthopaedic injury was ACL injury in skiers [6, 16]. Similar to the study by Posch et al. [20], all analysed knee injuries in their study involved an ACL injury, and the ACL is predominantly injured in all knee injuries needing hospitalization. Moreover, regarding sex differences in ACL injuries, the present study found that the proportion of the ACL injury among knee injuries was 1.4 times higher in female skiers than in male skiers. This result was consistent with previous studies that reported that females had a higher ACL injury rate than males [15, 28]. In contrast, some other studies reported that no significant sex differences were observed in the ACL injury rate [16–18]. Since these studies did not distinguish between single ACL injury and ACL combined with other ligament injuries, it is difficult to compare these results with the results of the current study.

The results of this study showed that males were taller, heavier, and with a higher BMI than females. These results are consistent with the findings by Burtscher et al. [29], who also observed higher body mass index and taller height in male skiers than in females. It is well-known that a massive body is beneficial in alpine skiing because it helps overcome frictional forces through a higher amount of potential energy [30], which may explain why there is a rising trend in body weight among elite alpine skiers [31]. Vermeulen et al. also found that additional fat tissue in both sexes may be beneficial in SPEED events during alpine skiing [32]. Posch et al. also reported that ACL-injured skiers had less weight and a lower BMI than non-injured skiers [33]. As the results of this study also found that females demonstrated a higher proportion of ACL injuries, body weight and BMI potentially play an important role in the high risk for ACL injuries in female skiers compared to male skiers. Since this study is only a cross-sectional study, it cannot be determined whether body weight is an injury risk factor. The relationships between the body weight and the ACL injury risk need to be further investigated by comparing the injured and uninjured groups.

The results of this study support our hypothesis that female skiers would have a significantly lower proportion of the multiple knee ligament injuries among knee injuries than male skiers. The current study demonstrated that the proportion of the multiple ligament injuries among knee injuries was 2.7 times higher in male skiers than in female skiers. The present study also showed that the combined ACL and PCL injury was not observed among female skiers, but four cases were found with combined ACL and PCL injuries and six cases were found with combined ACL, PCL, and MCL injuries among male skiers. Since the anterior and posterior cruciate ligaments are considered to be significantly responsible for ensuring stability, simultaneous ACL and PCL ruptures are very severe injuries resulting in distinct instability of the knee joint followed by early degenerative arthritis [34, 35]. The higher proportion of severe injury among men than women could be related to different skiing techniques and strategies. Another possible explanation for this difference might be risk-taking behaviours. In recreational skiing, it is suggested that men are more prone to challenge themselves through risky behaviours than women [36]. Although no researcher, for now, compared the vertical drop and skiing speed between sexes in recreational skiers, men usually compete with a longer and higher vertical drop and higher skiing speed in racer skiers [6]. A high vertical drop usually results in high speed, which could cause high kinetic energy, thus severe injuries. A recent systematic review demonstrated that large drops to the ground have higher injury odds and rates, and are risk factors for severe ski injuries [37]. The results of the current study combined with the literature suggest that choosing a proper skiing behaviour based on the physical fitness and skill level might be essential to prevent severe injuries for men.

The results of this study supported our hypothesis that significant sex differences will be observed in the physical activity habits among the injured skiers. The higher proportion of skiers with vigorous-intensity activity level in males indicated that male skiers have a higher overall level of exercise than female skiers. The physical activity habits in daily life may have an influence on the lower extremity injuries. Since the skiing activity is a high-intensity exercise, the individuals who do not often engage in a high-intensity exercise in daily life might be at high risk for injury when they are skiing. The vigorous-intensity activity habits in daily life might assist skiers improving the physical fitness, and increased intensity in daily physical activity might be beneficial in reducing risk injury during alpine skiing. Future studies are needed to explore more deeply about the relationship between different levels of physical activity and ski injury.

This study demonstrated detailed knee injury patterns among recreational skiers, which provided crucial theoretical evidence supporting sex differences in knee injury patterns among recreational skiers and set a basis for future studies in this area. Furthermore, the present study results suggested that the difference in physical activity habits between female and male skiers might influence differences in the injury patterns between sexes.

The present study analysed the injury characteristics at a single institute, and only individuals undergoing surgical intervention are included in the study. Therefore, there is a high probability of under-reporting injury rates, limiting its external validity. However, this study provides a precise diagnosis of each patient analysed in this study instead of injury registration through retrospective interviews with skiers or medical staff. Particularized injuries cannot be differentiated because of coding limitations in the registration system [38]. Also, the results of the present study only showed that injury patterns differ between sexes. Further studies are needed to investigate the sex-specific risk factors of the different injury patterns. Finally, this study is a cross-sectional reporting study. Longitudinal prospective studies are needed in the future to determine the risk factor of skiing injury.

## Conclusion

Anterior cruciate ligament injury is the most common orthopaedic injury among both female and male knee-injured recreational skiers. The proportion of anterior cruciate ligament injury of female skiers is higher than that of male skiers. The proportion of multiple ligament injuries among knee injuries is also higher in male skiers than among female skiers. More male recreational skiers have vigorous-intensity activity level habits in daily life than females.

## Data Availability

The data generated and analysed in the present study are not publicly available due to ethical restrictions. However, the data are available from the corresponding author on reasonable request.

## Abbreviations

ACL: Anterior cruciate ligament
BMI: Body mass index
MCL: Medial collateral ligament
PCL: Posterior cruciate ligament
LCL: Lateral collateral ligament
MPFL: Medial patello-femoral ligament
